# Postmortem CT analysis of paranasal sinuses using an experimental model of drowning

**DOI:** 10.1007/s00414-024-03173-8

**Published:** 2024-02-13

**Authors:** Alexander Tyr, Brita Zilg, Tobias Gelius, Rasmus Möllby, Nina Heldring

**Affiliations:** 1 Swedish National Board of Forensic Medicine, Retzius v. 5, 171 65 Stockholm, Sweden; 2 Department of Oncology-Pathology, Karolinska Institutet, Retzius v. 3, 171 77 Stockholm, Sweden

**Keywords:** Paranasal sinuses, Forensic pathology, Autopsy, Postmortem imaging, Immersion, Passive fluid influx

## Abstract

Fluid-filled paranasal sinuses are suggested to be a valuable tool to distinguish between drowning and non-drowning postmortem, yet the mechanisms governing fluid entry remains unknown. We investigate if fluid-filled paranasal sinuses are caused by a passive influx from submersion or an active aspiration mechanism during drowning. The ovine nasal cavity and maxillary sinuses are remarkably similar anatomically to humans, and have been used for endoscopic surgical training in recent decades. We submerged 15 decapitated ovine heads from agricultural waste at a depth of 2 m in flowing water for 1, 8, and 24 h and 7 days. Paranasal sinuses were CT imaged and compared pre- and post-submersion to non-submerged controls. Furthermore, we examined the paranasal sinuses of a single homicide case of a non-drowned submerged subject. Results demonstrate that fluid passively enters the maxillary sinus postmortem in the non-drowned ovine heads following 1 h of submersion. Fluid volume was independent of submersion time and influenced by time out of water as well as handling, since volume was reduced between consecutive CT scans. In contrast to our hypothesis, the filling of the paranasal sinuses is due to passive influx of fluid from submersion rather than an active aspiration during drowning. The observation that paranasal sinuses were fluid-filled in a single medico-legal case of postmortem submersion supports the finding of passive influx. Consequently, careful interpretation of fluid-filled paranasal sinuses is required when bodies are found in water, as the finding cannot distinguish between postmortem submersion and drowning.

## Introduction

Despite being a major cause of unintentional death worldwide, diagnosing drowning postmortem remains challenging. This is largely due to the complex pathophysiology that occurs during drowning resulting in autopsy findings difficult to interpret. Fluid-filled paranasal sinuses are suggested to be a valuable tool to differentiate between drowning and non-drowning [1], yet the mechanisms governing fluid entry into the sinuses remain unknown. In this study, we seek to investigate if fluid-filled sinuses are caused by a passive influx from submersion or require an active aspiration mechanism that occurs during drowning.

To determine drowning as a cause of death, an array of pathological indicators may be examined during autopsy. This includes macroscopic findings, such as foam in the respiratory tract [2–5], pulmonary edema and pulmonary emphysema [6–8], that may also be examined microscopically [9–11]. However, none of these traits are pathognomonic for drowning [12], and nor do all individuals presumed to have drowned exhibit all or any of the pathologies, rendering them unreliable. Biochemical changes in electrolyte balances have also been examined in a number of studies to provide alternatives for diagnosing drowning [13–16], but are limited by their dependence on variable factors such as aspiration volume and fluid salinity [17, 18]. Other findings such as neck and torso hemorrhages [19–21], sedimentation of gastric content, and organ size alterations [22–24] have also been suggested, although are as of yet inconclusive. The use of diatoms has also been deliberated in the literature, but are argued to be influenced by antemortem exposure, lacking reference values, and are prone to contamination [25–27]. Drowning is therefore a diagnosis of exclusion. Perhaps the greatest challenge facing postmortem drowning research is that drowning is difficult to determine independently of the diagnostic findings examined, leading to inadvertent circular fallacy.

As it is generally accepted that some degree of aspiration occurs during drowning [28, 29], it may be hypothesized that fluid is forced into the paranasal sinuses in the process [1]. The presence of fluid in the sphenoidal sinus of individuals presumed to have drowned is thought to have been initially described by Svechnikov in 1965 [30]. However, since the sinuses were aspirated blindly during autopsy, it is difficult to draw meaningful conclusions. The advent of powerful imaging technology, such as computer tomography (CT), has enabled a more robust and accurate approach to investigate fluid-filled paranasal sinuses [31], with several studies demonstrating that between 90 and 100% of suspected drowning victims exhibit the finding [5, 32–36]. On the other hand, so do a significant proportion of non-drowned victims, where the cause of death ranges from mechanical asphyxiation (hanging) to burn and fire deaths [32, 34, 35, 37]. No study to date has methodically explored the effects of water penetration into the sinuses of non-drowned subjects submerged postmortem.

**Fig. 1:**
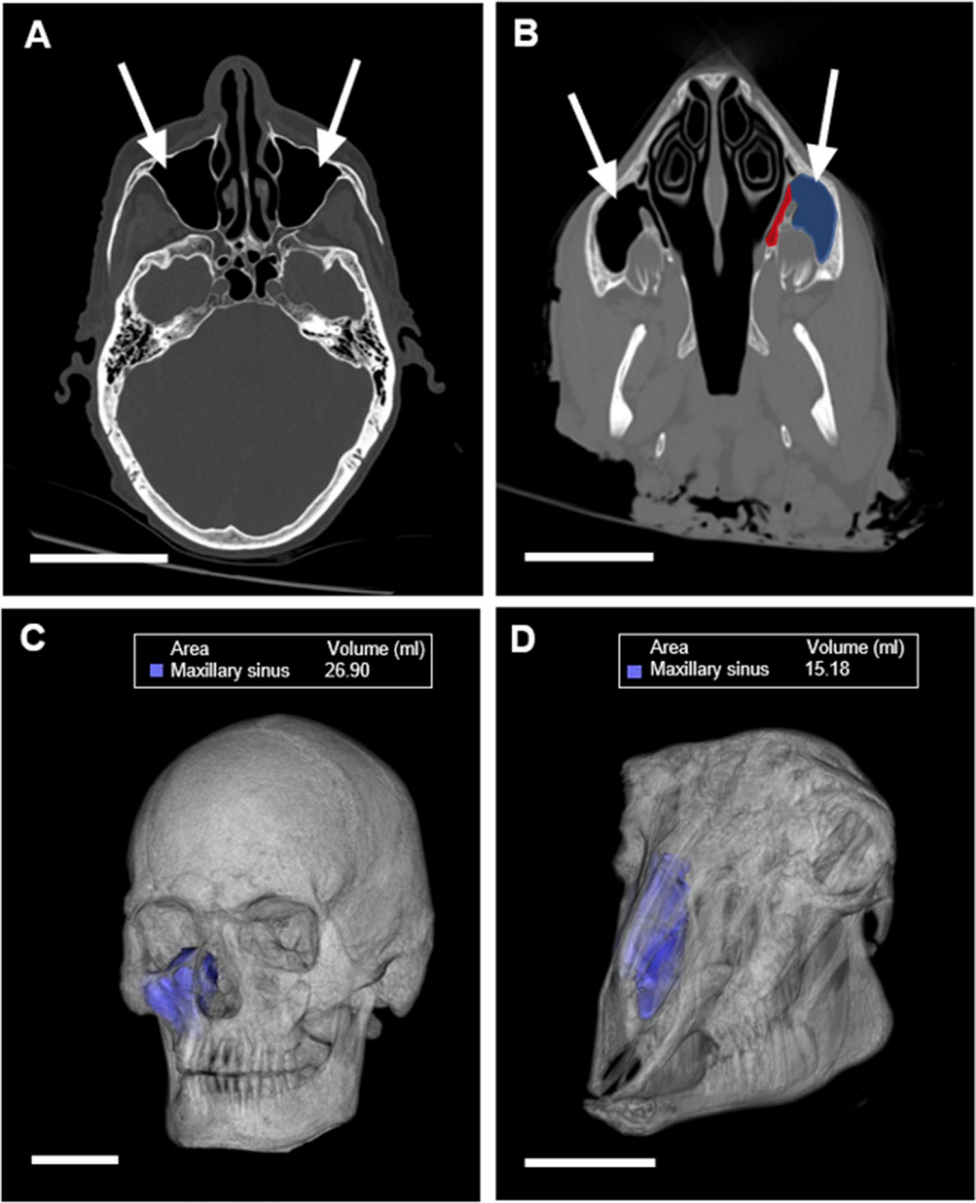
CT imaging of maxillary sinuses. CT imaging of left and right human (**A**) and ovine (**B**) maxillary sinus (white arrows) viewed in the axial plane. Red and blue renderings indicate medial and lateral aspects of sinus, respectively. 3D representation of human (**C**) and ovine (**D**) head from CT, where blue rendering indicates the volume of the right maxillary sinus. Scale bars = 5 cm

**Fig. 2:**
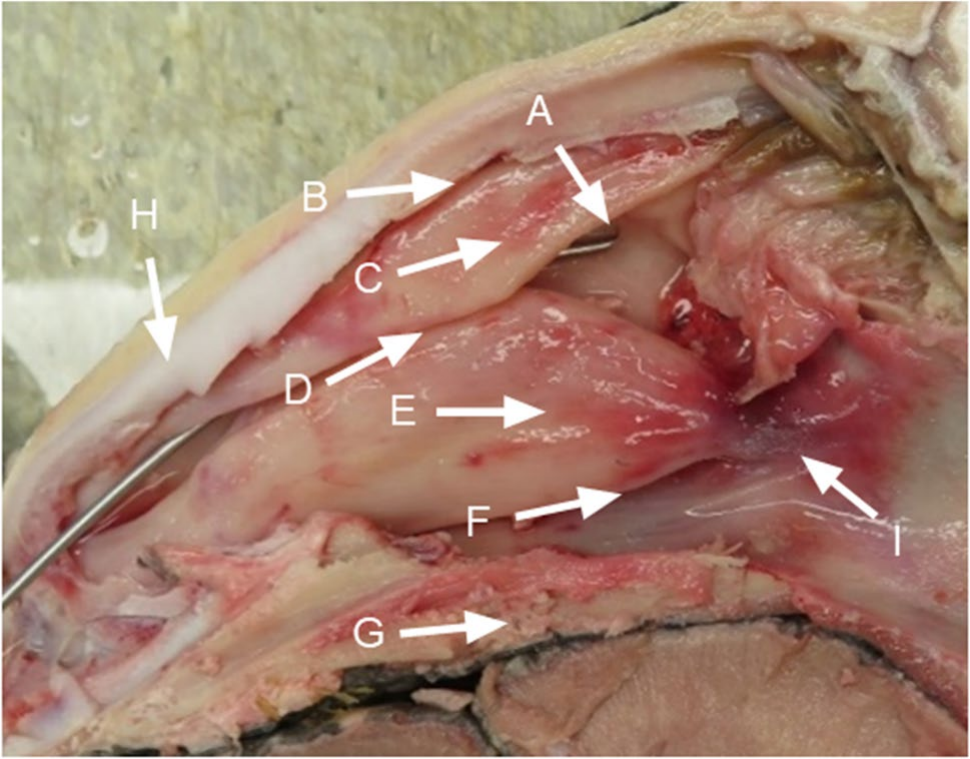
Ovine dissection of nasal cavity. Dissection of ovine head sectioned in the median plane. (A) Maxillary ostium with metal rod inserted into the maxillary sinus. Opening measured 1.5 mm in diameter with a length of approximately 2 cm. (B) Superior nasal meatus. (C) Superior dorsal concha. (D) Middle nasal meatus. (E) Middle dorsal and ventral spiral lamellae of the ventral nasal concha. (F) Inferior nasal meatus. (G) Hard plate. (H) Lateral process of nasal cartilage. (I) Choana

The ovine nasal cavity and paranasal sinuses are remarkably analogous anatomically to that of humans, in that they are also composed of distinct regions, including the frontal and maxillary sinuses (Fig. 1). Akin to that of humans, the maxillary sinus communicates with the nasal cavity via a single membranous ostium, similar in size and situated rostrally in the middle meatus between the superior and middle dorsal concha (Fig. 2). Indeed, notable differences in size and shape exist between the two species. For example, the frontal (conchofrontal) sinuses in sheep are located more cranially than in humans, and the maxillary sinuses are larger relative to the overall size of the skull that are structured into two regions (a medial and a larger lateral region) (Fig. 1B) [38]. Despite the absence of the sphenoid sinus in quadrupeds, the ovine nasal cavities are still regarded as valuable and suitable alternatives for endoscopic sinus surgical training [39–41].

The present study investigates if penetration of fluid into paranasal sinuses may occur postmortem in an ovine model. This approach allowed us to employ a cause-and-effect experimental design to address our question and to further determine if there is a link between volume and submersion time. It should be mentioned that the use of only heads as opposed to entire bodies should not affect results, since the fluid’s entry point into the sinuses remains unchanged. Furthermore, we analyze the relationship between fluid-filled sphenoidal and maxillary sinuses in humans and describe the paranasal sinuses in a single medico-legal case of homicide where the subject had been subsequently submerged postmortem. We hypothesize that fluid penetration into the paranasal sinuses requires an active aspiration mechanism and that fluid volume is time dependent. We therefore predict that no fluid will be observed in the maxillary sinuses following submersion during earlier time points.

## Methods

### Animals

Eighteen ovine heads aged between 8 and 14 months were kindly provided by HK SCAN Sweden, an established food manufacturer of meat products. Prior to study start, relevant permissions were obtained from the Swedish Board of Agriculture for the handling and disposal of the biomaterial. The study approach was also agreed with the Stockholm County Administrative board. Following planned animal sacrifice by HK SCAN Sweden (in accordance with the Swedish National Food Agency), the heads were immediately transported the Swedish National Board of Forensic Medicine where they were stored and cataloged prior to CT imaging. 

### Experimental procedure

**Fig. 3:**
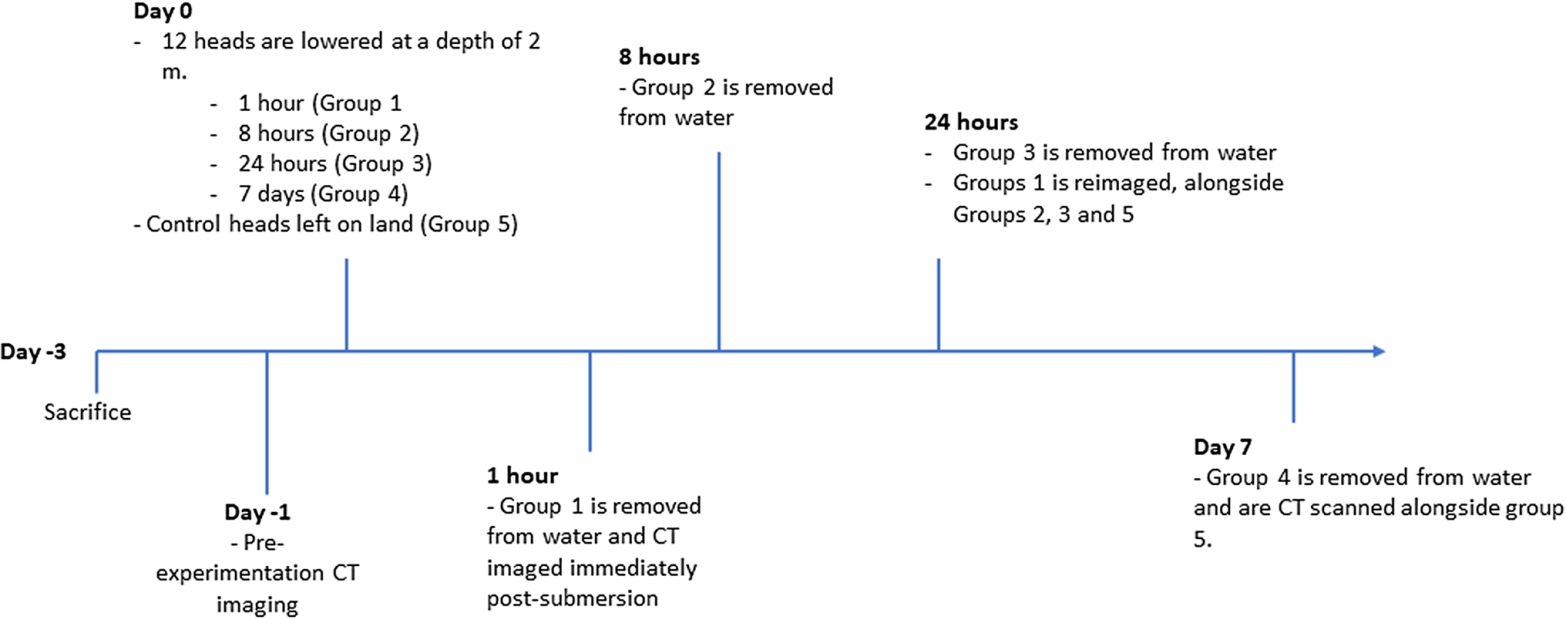
Experimental procedure scheme. Experimental scheme for ovine head submersion and CT imaging

CT imaging of ovine maxillary sinuses was conducted both pre- and post-submersion. Heads were divided into five test groups of three, each group subjected to a different experimental time condition (Fig. 3). Group 1 was subjected to a 1-h submersion; group 2, 12-h submersion; group 3, 24-h submersion; and finally group 4, 7-day submersion (168 h). All heads were submerged at a depth of 2 m, in brackish water with an average temperature of 11 °C. Heads in group 0 were used as negative controls that were stored on land at an average temperature of 12 °C and never subjected to submersion. CT imaging was conducted on group 1 immediately following submersion completion and again 24 h later alongside groups 2 and 3. CT imaging of group 0 and 4 occurred on experimental day 8. CT scans were acquired using a single source, 80 row detector, helical CT-scanner (Canon Aquilion Prime SP). Acquisition parameters: 120 kV, 580 mA, 1-s rotation time, collimation 0.5 × 80, detailed pitch. For image reconstruction, we used a noise reducing deep learning image reconstruction algorithm (AiCE), bone and body sharp kernels, 1.0-mm slice thickness, slice interval 0.8, and variable FoV to individually focus on the skull shape. No contrast medium was added. Image reading was performed using Vitrea Advanced Visualization (Canon Medical Systems) and was carried out by a single forensic pathologist with several years of postmortem imaging experience in humans. A random set of images were additionally assessed by an independent researcher also with postmortem imaging experience in humans, to control for differences in measuring.

### Retrospective investigation

In order to examine the frequency of fluid-filled maxillary and sphenoidal sinuses in human drownings, all witnessed fatal drownings in Sweden between 15 June 2021 and 30 September 2022 were extracted from the Swedish National Board of Forensic Medicine database, together with data on age, sex, drowning circumstances, and postmortem interval (PMI). From an initial list of 82 cases, 44 cases were included. Exclusion of 38 cases was based on hospital treatments (*n* = 7), poor CT quality (*n* = 1), other competing causes of death (*n* = 12), decomposed state (*n* = 12), and being below the age of 18 (*n* = 6).

### Non-drowned submerged control

CT scanning forms part of the routine medicolegal death investigation in Sweden. CT scans and measurements of paranasal sinuses together with circumstantial data were collected for one control individual who had been subject to a medicolegal investigation. Radiodensity was measured in Hounsfield units (HU) in order to attain a better understanding of putative fluid composition. The manner of death was concluded as homicide caused by fatal sharp-force trauma to body parts other than the head. The body had been subsequently submerged following death.

## Results

### Ovine investigation

**Fig. 4:**
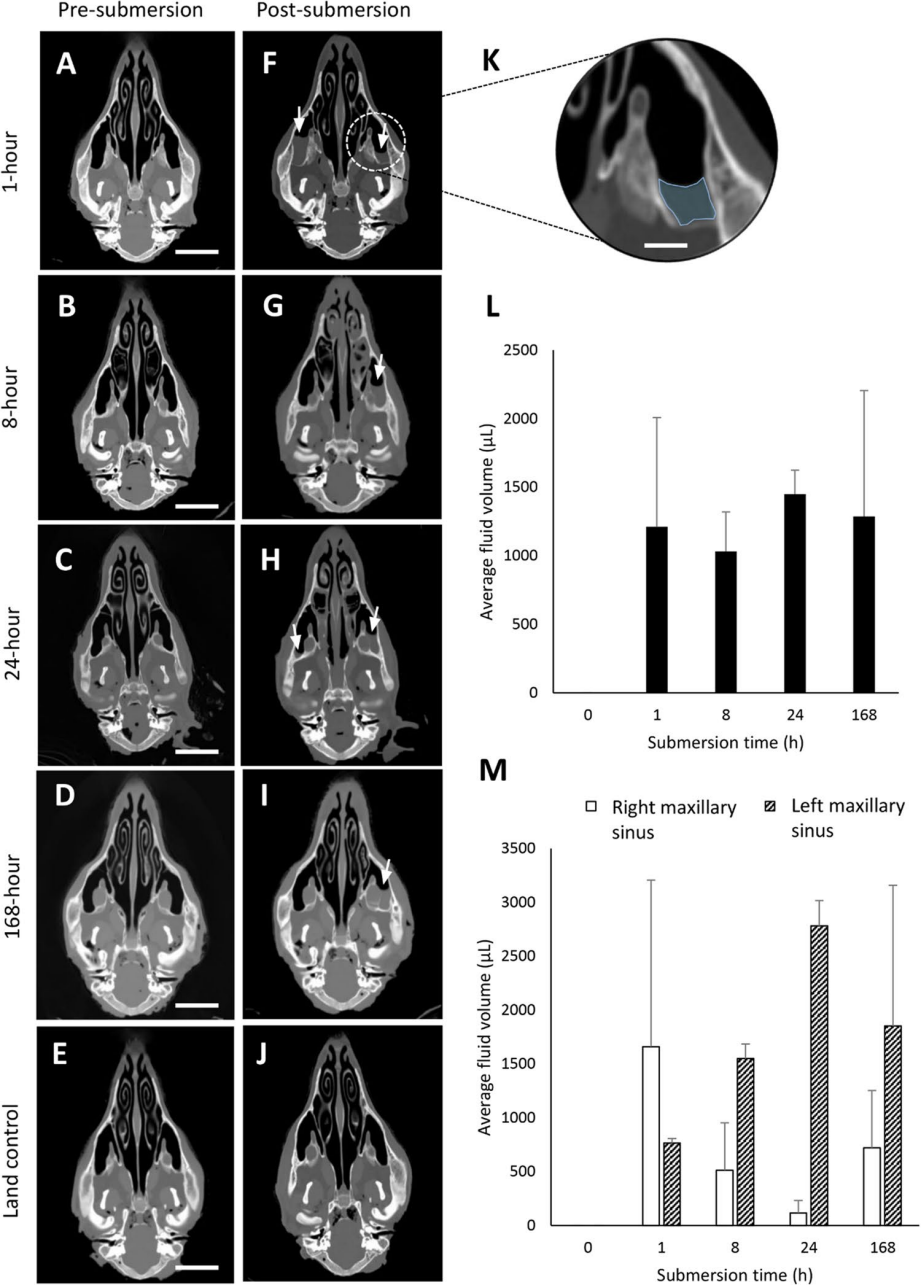
Fluid-filled maxillary sinuses postmortem. Ovine heads pre- (**A**–**E**) and post-submersion (**F**–**J**). Scale bar = 3 cm. Zoomed in region of left maxillary sinus with blue outline indicating fluid (**K**). Scale bar = 1 cm. Average fluid volume in both left and right maxillary sinus in all test groups over time (**L**). Left and right average maxillary sinus fluid volumes in all 5 groups over time (**M**)

**Fig. 5:**
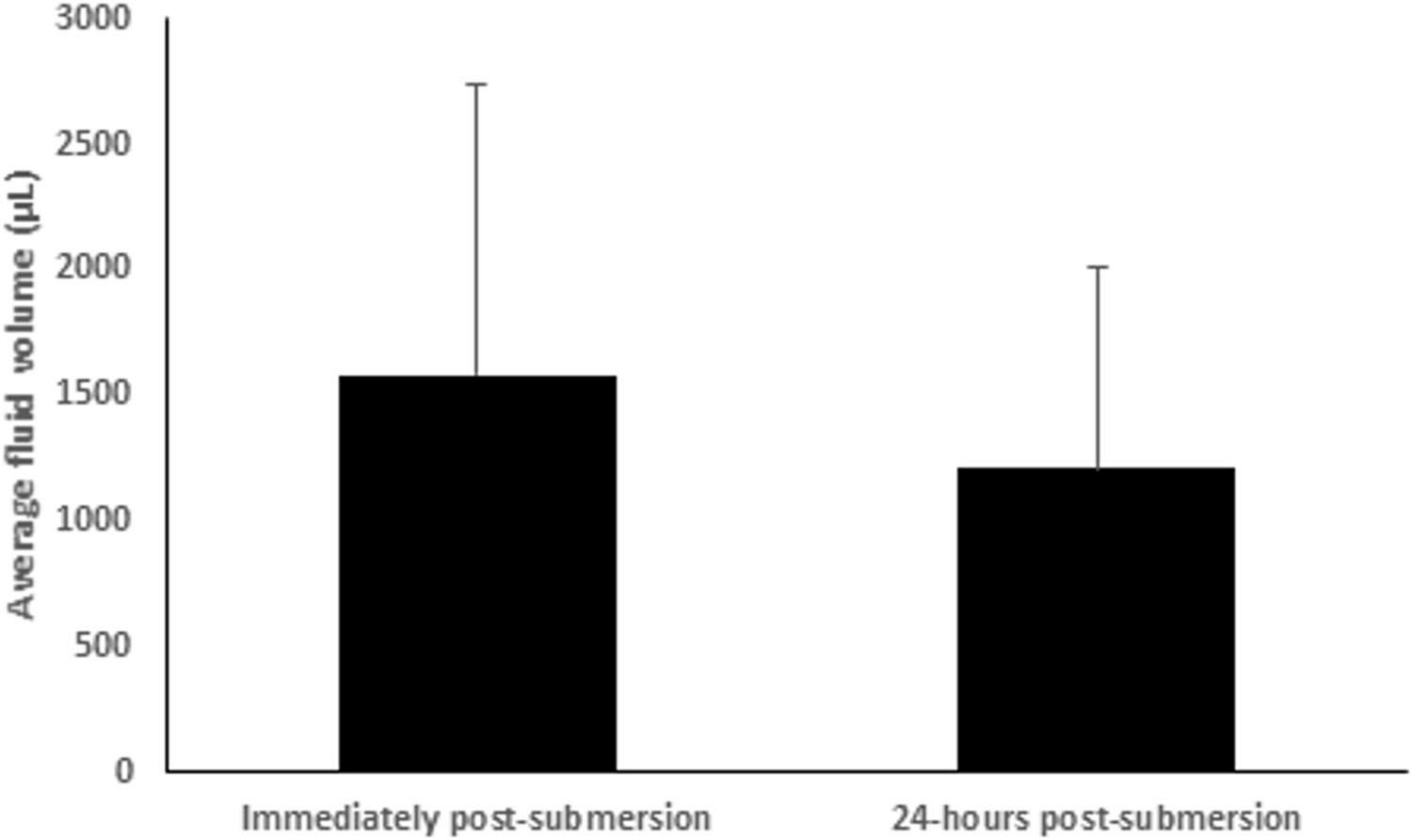
Average fluid volume immediately post-submersion and 1-day post-submersion. Average total fluid from maxillary sinuses of group 1 heads immediately post-submersion and 24-h post-submersion, demonstrating a 23% reduction in average fluid volume

No fluid was observed in the maxillary sinuses during pre-submersion imaging or in land control groups. Fluid was observed in the maxillary sinuses of all submerged ovine groups with an average volume of 1.25 ml (Fig. 4). Similar volumes were noted between all groups, indicating that submersion time had no effect on volume. Furthermore, fluid volume differences between the left and right demonstrated that left sinus had a significantly greater volume than the right (1.74 ml vs 0.75 ml, respectively) at all but one time point (1 h) (Fig. 4M). In the heads imaged immediately post-submersion and again 24-h post-submersion (Fig. 5), a 23.4% decrease in volume was demonstrated (1.58 ml vs 1.21 ml, respectively).

### Retrospective investigation

All but one of the 44 witnessed cases in the retrospective investigation had fluid in their paranasal sinuses. The frequency of fluid-filled sphenoidal sinuses was 86% and the frequency of fluid-filled maxillary sinuses was 98%. Neither frontal nor ethmoidal sinuses were examined.

### Non-drowned submerged case

**Fig. 6:**
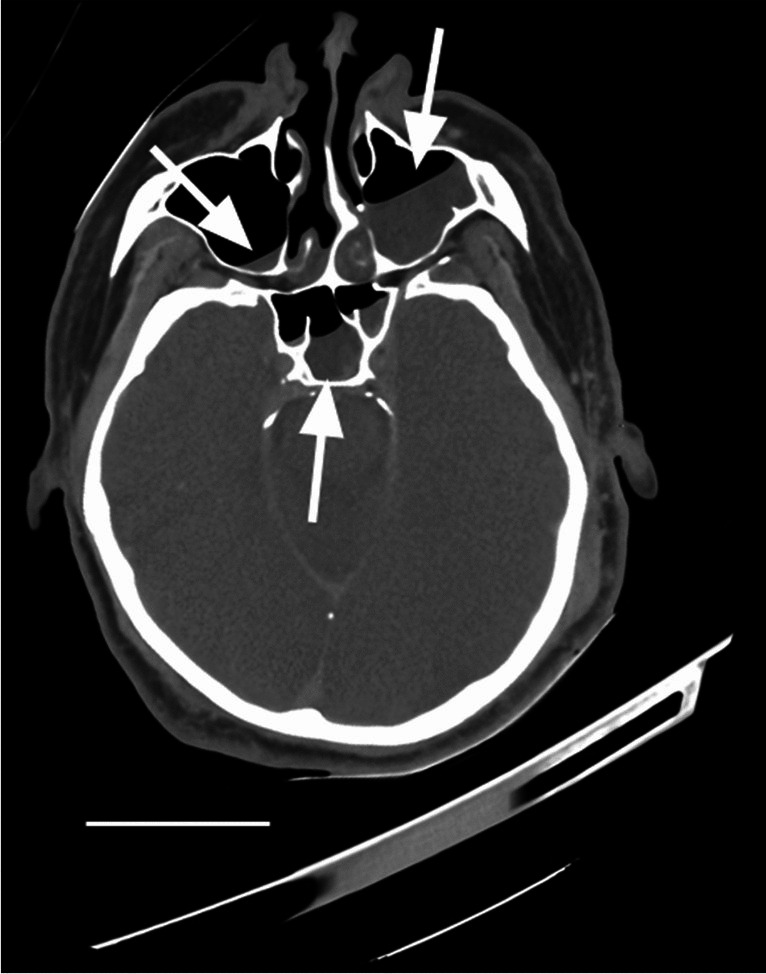
Non-drowned submerged human. Representative image of CT scan of non-drowned human submerged following homicide. White arrows indicate fluid in both maxillary sinuses and sphenoidal. Scale bar = 6 cm

With regard to the non-drowned submerged human case, results demonstrate that both maxillary sinuses as well as the sphenoidal sinus contained fluid (Fig. 6 and Table 1). The left maxillary sinus contained a total of 10.5 ml with a HU of 16.8 ± 6.6, while the right contained 0.3 ml of fluid (HU, 18.6 ± 7.6). The sphenoidal sinus exhibited 2.3 ml of fluid with a HU of 23.0 ± 9.3.

**Table 1:** Paranasal sinus measurements of non-drowned human submersion

	Volume (ml)	HU
Left maxillary sinus	10.5 ml	16.8 ± 6.6
Right maxillary sinus	0.3 ml	18.6 ± 7.6
Sphenoidal sinus	2.3 ml	23.0 ± 9.3

## Discussion

Results demonstrate that fluid enters the maxillary sinus postmortem in non-drowned ovine heads, following a 1-h submersion at a depth of 2 m and that fluid volume is independent of submersion time. Further analysis suggests that volume may be influenced by time out of water and handling as volume was reduced between CT scans in one of the submerged groups. In contrast to our hypothesis, the filling of the paranasal sinuses in an ovine model is due to the passive influx of fluid from submersion, and is not dependent on an active aspiration during drowning. This is supported by the discovery of fluid-filled paranasal sinuses in the case of a non-drowned individual, who was first killed and subsequently disposed of at a depth of 2 m for approximately 4 days.

Results from this study challenge the recent meta-analysis and systematic review by Da Trindade et al. [1], who conclude that fluid-filled paranasal sinuses may be used for the diagnosis of drowning. However, the meta-analysis does not address the pertinent issue of passive influx of water as a consequence of submersion, stating that the number of submerged bodies in the control groups examined were not detailed in most cases [1]. It may be argued therefore, that the studies selected by Da Trindade et al. [1] are of limited value, as the comparison between drowning victims and non-submerged land-based controls is debatable. In effect, these studies do not answer the question if fluid-filled paranasal sinuses are a pathological finding following drowning, but rather that they are not following death by an alternative cause of a death. While valuable in various forensic scenarios, it is essential to emphasize that until submerged non-drowned controls are thoroughly investigated, utilizing fluid-filled paranasal sinuses as a diagnostic tool to differentiate between drowning and non-drowning cases should be approached with caution.

Our study raises a critical question regarding which paranasal sinuses are required to be fluid-filled for the finding to be classified as positive? Initially, sphenoidal sinuses were considered as the hallmark most due their accessibility during autopsy. The description of fluid-filled paranasal sinuses is also misleading, as the term encompasses both ethmoidal and frontal sinuses in addition to the maxillary and sphenoidal that have not been studied. Our retrospective investigation revealed that 87% of witnessed drownings exhibited fluid-filled sphenoidal sinuses, whereas the frequency of fluid-filled maxillary sinuses was 97%. If this difference is due to variations in filling mechanisms, anatomy or handling effects remain to be determined.

Anatomical differences among sinus openings could potentially influence the entry of fluid, and therefore, the absence of fluid in the sinuses may not necessarily rule out drowning as a cause of death in bodies found in water. While the ovine model has limitations in that the sinus sizes differ somewhat to that of humans [39], they provide a unique and valuable opportunity to investigate the mechanistic insights into sinus pathophysiology [41, 42]. The rotation of heads during examination was also noted to cause a reduction in volume and it may be further hypothesized that the volume differences between sinuses in our study may be due to their position during submersion. The ovine heads were placed at random in either a supine or prone position in the submerged netting. This may explain similar findings in the case of the non-drowned submerged human that demonstrated a significant difference between left and right maxillary sinuses (Table 1). Examination of fluid volume immediately post-submersion vs 24-h post-submersion also demonstrated a 23% reduction in volume. This highlights that positional changes during the collection, transportation, and examination of a body may influence fluid volume, reducing its reliability as a marker.

Nevertheless, it is important to note that the ovine model cannot definitively dismiss the possibility that fluid-filled sinuses may still be indicative of drowning in humans. Although CT has become the new benchmark for analyzing fluid-filled paranasal sinuses, there is currently no standardized approach for conducting such forensic examinations. Vander Plaetsen et al. [33] categorized sinus fluid into three analysis groups based on the volume occupied space relative to free space, measured in mm, while Van Hoyweghen et al. [34] used a more rudimentary binary approach (present vs non-present). The use of such a binary analysis scheme may account for the substantially high number of non-drowned victims that exhibit fluid in their sinuses [31, 32, 34, 35, 37]. This overlooks the possibility that drowning victims may experience a greater influx of fluid compared to non-drowning victims and that fluid composition may differ. Analysis of fluid density has been conducted in the literature, but reference values remain unknown due to significant variation between studies [37, 43, 44]. In the present study, fluid volume was measured in mm^3^ and expressed as ml, suggested to be more meaningful. No differences in sinus fluid volume following salt vs freshwater have been reported in the literature, although a higher fluid density following saltwater immersion has [45].

In conclusion, the present study provides novel insights into fluid-filled paranasal sinuses. Postmortem submersion using an ovine model at a depth of 2 m demonstrated to passively fill the maxillary sinuses regardless of submersion time and that post-submersion handling and storage reduces the volume. Our results indicate that fluid-filled paranasal sinuses are markers of submersion and challenge the reliability of fluid-filled paranasal sinuses as a conclusive marker for drowning. Previous studies have not compared drowning populations to non-drowned submerged controls and we highlight the need for future studies to address this and to standardize analysis from CT images. While we acknowledge that the use of an ovine model cannot definitely disprove fluid-filled paranasal sinuses as a drowning sign in humans, our findings underscore the importance of cautious interpretation when bodies are found in water. Since the underlying biological drowning mechanisms and anatomical variations governing fluid entry during drowning continues to remains elusive, we conclude that fluid-filled sinuses should as of current be considered markers of submersion, not drowning.
